# The short term burden of ambient fine particulate matter on chronic obstructive pulmonary disease in Ningbo, China

**DOI:** 10.1186/s12940-017-0253-1

**Published:** 2017-06-06

**Authors:** Guoxing Li, Jing Huang, Guozhang Xu, Xiaochuan Pan, Xujun Qian, Jiaying Xu, Yan Zhao, Tao Zhang, Qichen Liu, Xinbiao Guo, Tianfeng He

**Affiliations:** 1 Department of Occupational and Environmental Health Sciences, Peking University School of Public Health, 38 Xueyuan Road, Beijing, 100191 China; 2 Ningbo Municipal Center for Disease Control and Prevention, Haishu District, 237 Yongfeng Road, Ningbo, 315010 China; 3 Ningbo First Hospital, 59 Liuting Street, Ningbo, 315010 China; 4 Tulan University, 6823 St. Charles Avenue, New Orleans, LA 70118 USA

**Keywords:** Fine particulate matter (PM_2.5_), Years of life lost, Chronic obstructive pulmonary disease, Modifications, Exposure-response curve

## Abstract

**Background:**

Numerous studies have found associations between ambient fine particulate matter (PM_2.5_) and increased mortality risk. However, little evidence is available on associations between PM_2.5_ and years of life lost (YLL). We aimed to estimate the YLL due to chronic obstructive pulmonary disease (COPD) mortality related to ambient PM_2.5_ exposure.

**Methods:**

A time-series study was conducted based on the data on air pollutants, meteorological conditions and 18,472 registered COPD deaths in Ningbo, China, 2011–2015. The effects of PM_2.5_ on YLL and daily death of COPD were estimated, after controlling long term trend, meteorological index and other confounders.

**Results:**

The impact of PM_2.5_ on YLL due to COPD lasted for 5 days (lag 0–4). Per 10 μg/m^3^ increase in PM_2.5_ was associated with 0.91 (95%CI: 0.16, 1.66) years increase in YLL. The excess YLL of COPD mortality were 8206 years, and 0.38 day per person in Ningbo from 2011 to 2015. The exposure-response curve of PM_2.5_ and YLL due to COPD showed a non-linear pattern, with relatively steep at low levels and flattened out at higher exposures.. Furthermore, the effects were significantly higher in the elderly than those in the younger.

**Conclusions:**

Our findings explored burden of PM_2.5_ on YLL due to COPD and highlight the importance and urgency of ambient PM_2.5_ pollution control and protection of the vulnerable populations.

**Electronic supplementary material:**

The online version of this article (doi:10.1186/s12940-017-0253-1) contains supplementary material, which is available to authorized users.

## Background

Ambient fine particulate matter (PM_2.5_) is one of the most important environmental issues worldwide, and the adverse impact of PM_2.5_ has become a great concern. Many epidemiological studies have provided evidences of associations between PM_2.5_ exposure and excess mortality [1–3]. The majority of these studies had estimated excess mortality using daily death counts as the dependent variable. However, using this indicator might ignore the differences in ages of deaths [4]. From a public health perspective, deaths occurring at different ages are not equally important.

Years of life lost (YLL) is a measure of disease burden that considers the life expectancy at death and, therefore, assigns higher weights to deaths that occur at younger ages [5]. Besides, it is more accurate than mortality to measure premature death and excess mortality [6]. In this sense, exploring the relationships between PM_2.5_ and YLL may provide additional information for policy making and health resource allocation, and has important public health significance.

Chronic obstructive pulmonary disease (COPD), characterized by irreversible airflow obstruction, is a common chronic respiratory disease [7]. Due to the slow progression and chronic nature of the disease, COPD represents a massive and growing disease burden and is an important cause of morbidity and mortality [8]. The Global Burden of Disease study showed that COPD was the third leading cause of deaths in the world and ranked the ninth of global YLL in 2010 [9]. There is compelling evidence that short-term exposure to particulate matter is a risk factor for the development and exacerbation of COPD [10–13]. However, the availability of studies on the associations between ambient PM_2.5_ and YLL of COPD is rare up to now, and the modifications of socioeconomic factors such as age, gender and marital status are unclear.

With the rapid development in industrialization and urbanization, ambient PM_2.5_ pollution has aggravated in China in recent years. Ningbo, located in the Yangtze River Delta in the southern China, is the world’s fourth-largest port city. The population in Ningbo was estimated to be 7.83 million in 2015, distributed in an area of approximately 9816 km^2^. The high level of economic development in the city also caused the ambient PM_2.5_ pollution to be a subject of highly concern.

In this study, we conducted a time-series study to explore the disease burden of COPD from short term ambient PM_2.5_ exposure in Ningbo, China, 2011–2015, using the indicator of YLL..

## Methods

### Data on mortality and YLL

Daily mortality data on deaths of COPD between January 1st 2011 and December 31th 2015 were obtained from Ningbo Municipal Center for Disease Control and Prevention, and the data was restricted to registered residents only. COPD deaths were identified according to the International Classification of Diseases 10th version (ICD-10: J40–44). The dataset comprised date of death, gender, age and marital status. Daily death count was defined as the number of deaths occurring on a single day.

We calculated YLL by matching the patient’s age to the life table for each death. The sex- and age-group specific life expectancy of Chinese population was obtained from the World Health Organization (WHO), and life expectancies for 2011–2015 were averaged (Additional file 1: Table S1). Daily YLL were calculated by summing the YLL for all deaths on the same day. Both daily YLL and deaths counts were stratified by age (<75 and ≥75 years), gender (male and female) and marital status (the married and the widowed).

### Exposure assessment

Concentrations of PM_2.5_, inhalable particulate matter (PM_10_), sulfur dioxide (SO_2_) and nitrogen dioxide (NO_2_) were monitored continuously at 11 monitoring sites which cover urban and suburban areas of Ningbo. The concentrations of the air pollutants were measured according to the Chinese National Ambient Air Quality Standard [14]. The Environment Monitoring Center of Ningbo collects the pollutants concentrations from all the monitoring sites of the city and records the hourly concentrations, from which the daily average levels for all the individual monitoring sites and the whole city levels were derived. Number of missing is less than 1%, and the missing values were substituted with the mean daily value. Daily meteorological data, including temperature and relative humidity, were obtained from the Ningbo Meteorological Bureau.

### Statistical analysis

We used generalized additive model (GAM) to explore the impacts of particulate matter on YLL. Because the dependent variable of daily YLL follows a normally distributed (Additional file 1: Figure S1), the family function for GAM was Gaussian.

The model is as follows:

$$ \mathrm{YLLt}=\alpha +{\sum}_{i=1}^q\upbeta \mathrm{i}\left(\mathrm{Xi}\right)+{\sum}_{j=1}^p\mathrm{fj}\left(\mathrm{Cj},\mathrm{df}\right)+\mathrm{Wt}\left(\mathrm{week}\right) $$

In the model, *YLL*_*t*_ is the observed daily YLL at day *t*; *α* is the intercept; *β* is the coefficients of YLL associated with per 1 μg/m^3^ increase in particulate matter; *Xi* is the daily mean concentrations of particulate matter; *Cj* is the confounding factors including time, daily temperature, daily relative humidity; *f*_*j*_ is the smooth functions (natural cubic spline); *W*_*t*_(week) is the dummy variables for day of week on day *t*. We applied the penalized spline function of time and 7 degrees of freedom per year was used to control for secular trend and seasonality. Degrees of freedom for temperature and relative humidity were set to 3 according to the previous studies [15, 16]. Considering the lagging effects of temperature, the 14-day moving average of temperature was used. For relative humidity, the average value of the present day was used in the models.

Then, to investigate the lag pattern of PM_2.5_, potentially delayed and cumulative associations were estimated. We first examined the delayed associations using a single day lag (from lag0 to lag7), as previous studies showed the lag effects of particulate matter were strongest within 7 days [2, 17]. Then the cumulative associations were estimated using the moving average over the lag periods from lag01 (moving average concentrations of day0 and day1) to lag07 (moving average concentrations of day0 to day7). Final results were presented as changes in daily YLL with per 10 μg/m^3^ increase in PM_2.5_ in different lag days. Considering the PM_2.5_ effect of 5-day moving average concentrations from day 0 to day 4 (lag04) is strongest, so we use lag04 in our main analysis.

We also calculated the excess YLL as follows:

$$ \mathrm{YLL}\ \mathrm{of}\ \mathrm{COPD}\ \mathrm{advanced}\ \mathrm{by}\ \mathrm{PMt}=\sum_{t=1}^{1826}\mathrm{PMt}\times \upbeta $$

Where PMt is the concentrations of PM_2.5_ (lag04) in our main studies at day t, *β* is the value of 1 μg/m^3^ particulate matter increase.

Secondly, we used the penalized spline of PM_2.5_ to replace the linear variable of PM_2.5_ to plot the dose-response curve of PM_2.5_ and YLL (Fig. 3). The relationship appeared to be nonlinear and the curve became flattening in high level PM_2.5_ concentration days, so we detected the turning point. The Akaike Information Criterion (AIC) values of GAM models using 1 unit increment in PM_2.5_ within the identified range of threshold were computed between 100 μg/m^3^ and 150 μg/m^3^(Additional file 1: Table S2). The concentration of PM_2.5_ with the lowest AIC value was selected as the turning point. Similar method had been used in previous studies [18–20]. According to the turning point (128 μg/m^3^), we created two variables (high concentration level, low concentration level), which were higher or lower than the turning point respectively, to explore the piecewise linear association of PM_2.5_.

In addition, the analyses were stratified by age, gender and marital status. Single pollutant model was used to explore the main association of PM_2.5_ with daily YLL, while two pollutants models with SO_2_ or NO_2_ added was used to examine the stability of these associations. In order to have a comparison, we also examined the effects of PM_10_.

Furthermore, we evaluated the impacts of particulate matter on daily death counts of COPD. The independent variable, lag structure and relevant degrees of freedom in the model were similar to those in YLL model, except time series function with the Poisson link under a GAM framework was used because the dependent variable daily death counts following a Poisson distribution. Results were presented as changes in excess risk (ER) of deaths from COPD per 10 μg/m^3^ increase in PM_2.5_ and PM_10_ in different lag days. The equation of ER was as follows:

$$ \mathrm{ER}=\left({e}^{10\times \beta}-1\right)\times 100\% $$

In the equation, *β* is the value of daily death counts associated with a unit increase in particulate matter.

An autocorrelation function (ACF) was used in the models to assess whether the residuals were independent over time and the reliability of the models. The results showed no obvious autocorrelations were found (Additional file 1: Figure S2 to Figure S5). Sensitivity analyses were performed on the parameters included in the models to test the robustness of our results. We tested using 4 of degrees of freedom of relative humidity, 4 of degrees of freedom of temperature, and used 6 & 8 of degrees of freedom of time per year in the models.

In this study, the mgcv packages in R software (Version 3.1.2) were used to perform the analyses. *P* < 0.05(2 side) were considered to be significant. The study was approved by the Institutional Review Board of Ningbo Municipal Center for Disease Control and Prevention (No. IRB 201603).

## Results

Mean daily concentrations of PM_2.5_ and PM_10_ were 49.58 μg/m^3^ and 78.59 μg/m^3^, respectively during 2011 to 2015 in Ningbo, China (Table 1). Correlations between air pollutants and meteorological conditions were examined by Spearman correlation function and presented in Table 2. Particulate and gaseous pollutant and meteorological conditions were correlated with each other, with the most significant correlations observed between the four air pollutants.

**Table 1:** Daily weather conditions, YLL and death counts of COPD in Ningbo, China, 2011–2015

Variables	Mean ± SD	Minimum	25th	Median	75th	Maximum
PM_2.5_ (μg/m^3^)	49.58 ± 32.36	5.86	28.25	41.73	61.25	421.71
PM_10_ (μg/m^3^)	78.59 ± 48.19	12.86	44.94	66.33	97.97	543.88
SO_2_ (μg/m^3^)	21.34 ± 13.84	5.38	12.14	16.94	25.73	112.6
NO_2_ (μg/m^3^)	43.41 ± 18.65	7.62	30.12	40.61	55	121.8
Temperature (°C)	17.49 ± 8.94	−1.67	10	18.89	24.44	34.44
Relative humidity (%)	76.41 ± 12.25	31	68	77	86	100
Years of life lost (years)						
Total	80 ± 40	0	51	73	102	278
Age < 75 years	19 ± 20	0	0	13	30	136
Age ≥ 75 years	61 ± 30	0	39	55	77	227
Male	46 ± 27	0	26	42	62	174
Female	34 ± 22	0	17	30	46	161
Married	44 ± 27	0	24	39	59	175
Widowed	33 ± 20	0	18	29	45	144
Daily death counts (No. of deaths)						
Total	10 ± 5	0	7	9	13	34
Age < 75 years	1 ± 1	0	0	1	2	8
Age ≥ 75 years	9 ± 4	0	6	8	11	33
Male	6 ± 3	0	3	5	7	18
Female	5 ± 3	0	3	4	6	20
Married	5 ± 3	0	3	4	7	19
Widowed	5 ± 3	0	3	5	7	20

**Table 2:** Spearman correlation between air pollutants and meteorological conditions in Ningbo, China, 2011–2015

	PM_2.5_	PM_10_	SO_2_	NO_2_	Temperature (°C)	Relative humidity (%)
PM_2.5_	1.00	0.95	0.78	0.76	−0.47	−0.28
PM_10_	0.95	1.00	0.83	0.76	−0.46	−0.42
SO_2_	0.78	0.83	1.00	0.79	−0.57	−0.42
NO_2_	0.76	0.76	0.79	1.00	−0.59	−0.13
Temperature (°C)	−0.47	−0.46	−0.57	−0.59	1.00	0.13
Relative humidity (%)	−0.28	−0.42	−0.42	−0.13	0.13	1.00

**Fig. 1:**
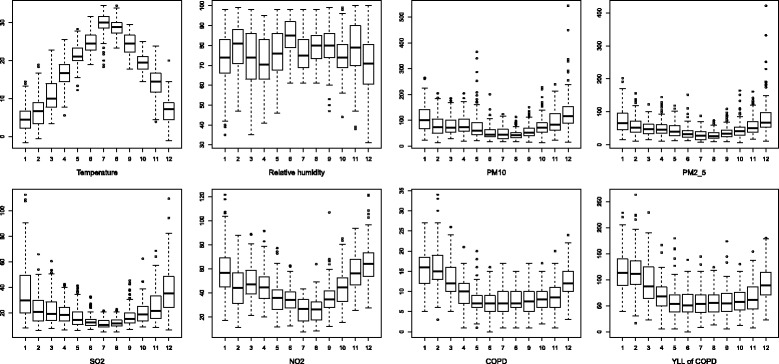
Boxplots of monthly PM_2.5_, PM_10_, SO_2_, NO_2_, temperature, relative humidity, years of life lost, death counts of COPD and the corresponding YLL in Ningbo, China, 2011–2015

A total of 18,472 registered COPD deaths were identified for the five study years. Daily death counts and YLL of COPD had a mean of 10 and 80 years, respectively. The average daily YLL of the elderly, male and the married ones were higher than that of the young, female and the widowed ones, respectively (Table 1). Both outcomes showed a seasonal trend, with higher values in November through the next April than other months (Fig. 1).

**Fig. 2:**
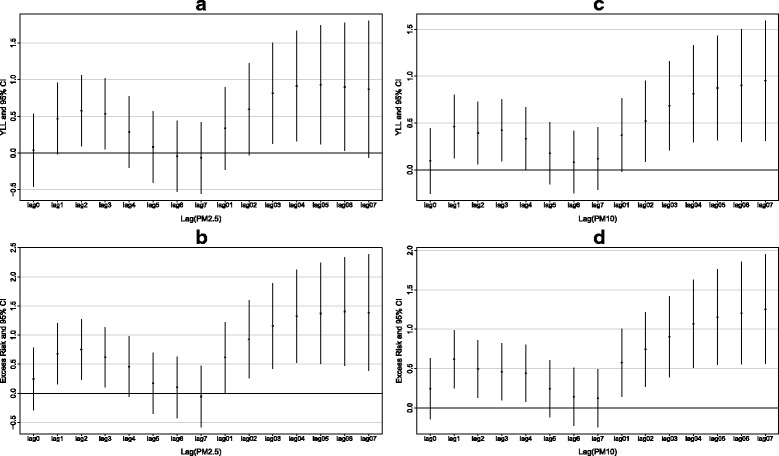
Estimated changes with 95% confidence interval in years of life lost and excess risk of deaths from chronic obstructive pulmonary disease associated with 10 μg/m^3^ increase in PM_2.5_ and PM_10_ in different lag days in Ningbo, China, 2011–2015. Results were adjusted for seasonality, day of the week, temperature and relative humidity (**a** Estimated changes in years of life lost associated with PM_2.5_; **b** Estimated changes in years of life lost associated with PM_10_; **c** Excess risk of deaths associated with PM_2.5_; **d** Excess risk of deaths associated with PM_10_)

Estimated changes with 95% confidence intervals in YLL due to COPD associated with 10 μg/m^3^ increase in PM_2.5_ in different lag days were presented in Fig. 2. In general, the associations with PM_2.5_ lasted for five days (lag0 to lag4), and gradually reduced after day 4. From day 5 to day 7, the effects had no significance. In addition, the lag patterns were similar for YLL and ER.

The strongest associations were found in 5-day moving average concentrations from day 0 to day 4 (lag04). In single pollutant model, a 10 μg/m^3^ increase in PM_2.5_ was associated with 0.91(95% CI: 0.16, 1.66) years increase in YLL, and 1.32% (95%CI: 0.52%, 2.12%) increase in ER for COPD deaths, respectively.

We also analyzed the associations of PM_10_ in our study. Per 10 μg/m^3^ increase in PM_10_ was associated with 0.81 (95%CI: 0.30, 1.33) years increase in YLL and 1.07% (95%CI: 0.51%, 1.63%) increases in ER of COPD death at lag04.

Furthermore, the excess YLL of COPD mortality in Ningbo from 2011 to 2015 were estimated, which were 8206 years. Take the population of Ningbo into consideration, the YLL in days per person of the population for a 365 day period of exposure was nearly 0.38 day per person in Ningbo from 2011 to 2015.

**Fig. 3:**
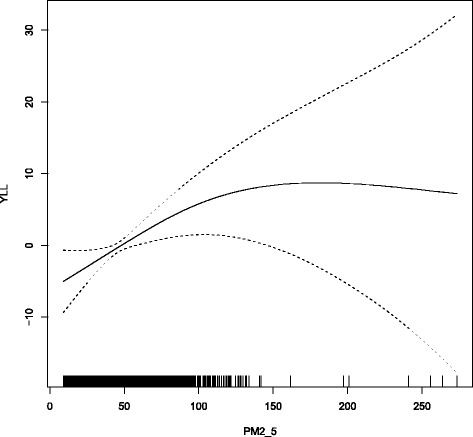
Exposure-response curve of the levels of PM_2.5_ and years of life lost from COPD in Ningbo, China, 2011–2015

In addition, the exposure-response curve of PM_2.5_ and YLL due to COPD showed a non-linear pattern (Fig. 3). When stratified by the turning point of PM_2.5_, the associations below the turning point concentration were stronger than those above the turning point concentration (Table 3). Specially, the associations of PM_2.5_-YLL due to COPD was 1.44 (95%CI: 0.57, 2.32) below the turning point concentration, while the value was 0.32 (95%CI:-0.59, 1.23) above the turning point concentration. And the mean age at death for PM_2.5_ concentrations above and below the median are 74.50 and 73.87, respectively. Similar pattern were showed for the associations between PM_2.5_ and ER of COPD deaths.

**Table 3:** Associations of 10 μg/m^3^ increase in PM_2.5_ and PM_10_ with YLL and ER of deaths from COPD

Pollutants and model	Years of life lost (95% CI)	Percentage increase in death (95% CI)
PM_2.5_ (Single model)		
Total	0.91(0.16, 1.66)	1.32(0.52,2.12)
Low concentration level	1.44(0.57, 2.32)	1.87(0.91, 2.83)
High concentration level	0.32(−0.59, 1.23)	0.84(−0.10, 1.79)
PM_2.5_ + SO_2_		
Total	1.28(0.46,2.11)	1.61(0.74,2.48)
Low concentration level	1.97(1.00, 2.93)	2.31(1.26, 3.37)
High concentration level	0.65(−0.30, 1.60)	1.12(0.14, 2.11)
PM_2.5_ + NO_2_		
Total	1.07(0.22,1.91)	1.53(0.64,2.42)
Low concentration level	1.45(0.45, 2.44)	1.98(0.97, 3.03)
High concentration level	0.39(−0.59, 1.36)	0.99(−0.05, 1.97)
PM_10_ (Single model)		
Total	0.81(0.30,1.33)	1.07(0.51,1.63)
Low concentration level	1.09(0.52, 1.66)	1.34(0.71,1.98)
High concentration level	0.35(−0.31, 1.01)	0.72(0.03, 1.41)
PM_10_ + SO_2_		
Total	1.08(0.51,1.65)	1.28(0.66,1.89)
Low concentration level	1.41(0.79, 2.04)	1.60(0.91, 2.29)
High concentration level	0.59(−0.09, 1.28)	0.91(0.19, 1.63)
PM_10_ + NO_2_		
Total	0.96(0.38,1.55)	1.26(0.63,1.89)
Low concentration level	1.18(0.53, 1.83)	1.51(0.82, 2.17)
High concentration level	0.46(−0.25, 1.17)	0.85(1.01, 1.63)

Associations in all concentrations and in low and high concentrations levels were both presented at lag04 (moving average concentrations from day 0 to day4). Single and two pollutants models were used. Data were collected from Ningbo, China, 2011–2015

In two pollutants models, the estimates for associations with PM_2.5_ and PM_10_ did not change much when the gaseous pollutants SO_2_ or NO_2_ were added in the models. For instance, when SO_2_ was added in the model, 10 μg/m^3^ increasing of PM_2.5_ was significantly associated with 1.28 (95%CI: 0.46, 2.11) person year increase in YLL.

The results of modifications of age, gender and marital status were summarized in Table 4. When stratified by age, the associations were stronger in the elderly (≥75 years) than the younger (<75 years). In the elderly, a 10 μg/m^3^ increase in PM_2.5_ corresponded to 0.98(95%CI: 0.42, 1.54) years increase in YLL. For the younger, no significant effects were found.

**Table 4:** Associations of PM_2.5_ with YLL and ER of COPD deaths stratified by age, gender and marital status

Indicator	Age		Gender		Marital status	
	<75 years	≥75 years	Male	Female	Married	Widowed
Years of life lost (95% CI)	−0.06 (−0.54,0.42)	0.98 (0.42,1.54)*	0.47 (−0.08,1.01)	0.45 (−0.04,0.94)	0.27 (−0.30,0.84)	0.63 (0.21,1.05)
Percentage increase in death (95% CI)	−0.05 (−2.39,2.30)	1.49 (0.65,2.33)*	1.12 (0.06,2.18)	1.57 (0.39,2.74)	0.84 (−0.31,2.00)	1.76 (0.66,2.86)

Associations were presented at lag04 (moving average concentrations from day 0 to day4) with 10 μg/m^3^ increase in PM_2.5._ Data were collected from Ningbo, China, 2011–2015. *P < 0.05

For the modification of gender, the associations between female and male were not significant. For different marital status, the associations in the widowed showed an increased trend compared with the married ones, though the effect estimate of the married was well within the widowed ones..

Sensitivity analyses results showed that the estimations were stable based on the variations of the parameters in the models (Table S4).

## Discussion

The relationships between ambient PM_2.5_ and increased mortality risk were well documented [1–3]. Although previous studies indicated that PM_2.5_ is a significant environment trigger for acute exacerbation of COPD, and thus leading to increasing symptoms, emergency visits, hospital admissions and mortality [10–13]. A study conducted in China showed that 83% of the population lived in areas where PM_2.5_ concentrations exceeded the Chinese Ambient Air Quality Standard of 35 μg/m^3^, premature mortalities attributed to PM_2.5_ nationwide were 0.17 million for COPD [21]. However, the number of death ignores the difference in ages of deaths, and giving equal weights to the deaths occurring at a young age and those occurring at an old age. From a public health perspective, deaths occurring at different ages are not equally important, dying at a young age results in more potential years of life lost (YLL) [4, 6]. Nevertheless few studies using YLL as dependent variable to explore the health impact of the current exposure levels of PM_2.5_ on COPD deaths in China, possibly due to the lack of individual data needed for calculating YLL.

Our study explored the associations of PM_2.5_ exposure with daily YLL due to COPD for the first time. Based on the data of 18,472 COPD deaths over a five year period in Ningbo, China, we found that PM_2.5_ exposure were significant associated with increased YLL of COPD. Per 10 μg/m^3^ increase in PM_2.5_ was associated with 0.91(95% CI: 0.16, 1.66) years increase in YLL of COPD deaths. In the study of He 2016 [5], an increase of 10 μg/m^3^ increase in PM_2.5_ was related to 2.97(95%CI: -2.01, 7.95) years of all-cause mortality, which using the data of Ningbo from 2009 to 2013. The comparison showed the impact of PM_2.5_ on YLL from COPD and all-cause mortality is different, although the impact of PM_2.5_ on YLL of all-cause mortality was not significant, there was significant influence on COPD YLL. This indicated more investigations should be carried out to explore the impact of PM_2.5_ on YLL from diseases which are leading cause of death and disease burden such as COPD.

In addition, the excess YLL of COPD mortality was also estimated, which were 8206 years. Take the population in Ningbo into consideration, the YLL in days per person of the population for a 365 day period of exposure was nearly 0.38 day per person from 2011 to 2015, which gives a different perspective from excess death number.

Considering the indicator of YLL is a measure of disease burden that considers the life expectancy at death [6], and has been extensively used to identify and prioritize causes of premature death around the world [22], the results of our study provides a complementary indicator to that of excess deaths, and gives additional information for policy making and health resource allocation.

The exposure-response curve of PM_2.5_ and YLL due to COPD showed a non-linear pattern in Ningbo, 2011–2015. The curve was relatively steep at low levels and flattened out at higher exposures. Previous studies also found similar pattern under different levels of particulate matter [20, 23, 24]. However, considering there were few observations for PM_2.5_, cautions should be taken when using the estimations in the high concentration level.

In this study, we found that the associations of PM_2.5_ on both daily YLL and death counts of COPD were stronger in the elderly (≥75 years) than the younger (<75 years). The difference was plausible from a biological perspective because of the decreased immunologic function and reduced lung function as a natural part of aging, and frailty status which decrease physiologic reserve for the adverse effects of air pollution, etc. [25, 26]. In addition, elderly individuals frequently have pre-existing chronic diseases, which may make them more vulnerable to the adverse influence of particulate matter exposure [27, 28]. The higher impact in the elderly was consistence with a previous study conduct in Ningbo which explored the air pollution-YLL relationships [5], while was different from the results from Beijing which indicated the influence of particulate matter on daily YLL was higher in the younger people [4]. Considering the annual concentrations of PM_2.5_ and PM_10_ were 105.1 μg/m^3^and 144.6 μg/m^3^ in the Beijing study, the difference may be due to the different levels of particulate matter in Beijing and Ningbo, which implied the associations of particulate matter and YLL would be different in various pollution levels.

For gender modification, although previous study indicated that female may be more susceptible to particulate matter pollution because female is a risk factor for developing airflow limitation and consequently COPD [29], furthermore, female with COPD would have worse prognosis due to more pronounced dyspnea, lower BMI, and more frequent anxiety [30], we did not found significant differences between gender.

It’s the first time to explore the potential effect modification of marital status in the particulate matter exposure and YLL relationships, though non significance was found. Further investigations still need to be carried out in future.

In our study, we also evaluated the particulate matter exposure and ER of death counts in our study. Our results showed a 10 μg/m^3^ increase in PM_2.5_ and PM_10_ were associated with maximum increases in ER of death counts of 1.32% (95%CI: 0.52%, 2.12%) and 1.07% (95%CI: 0.51%, 1.63%) at lag04, respectively. The results were supported by previous studies. For instance, meta-analysis of 31 studies showed that a 10 μg/m^3^ increase in PM_10_ was associated with increase in COPD mortality with an OR of 1.011 (95%CI: 1.008, 1.014) [31]. A study conducted in Guangzhou also found an increase of 10 μg/m^3^ increase in PM_10_ was associated with 1.58% (95%CI: 0.12%, 3.06%) increase of COPD mortality [19].

This study has several strengths. First, our study explored the associations between PM_2.5_ exposure and YLL due to COPD for the first time, and the excess YLL were also calculated. Using YLL as a key indicator to measure the impact of particulate matter on premature deaths will provide more information for policy making and resources allocation. Secondly, exploring the exposure-response curve of PM_2.5_ on YLL due to COPD was the first time. We found the exposure-response curve was nonlinear and the associations were steeper in the low PM_2.5_ concentrations and became flattening in the high PM_2.5_ concentrations. Thirdly, the modification of socioeconomic factors especially the marital status was first investigated, and provided complementary information for identifying vulnerable subgroups.

However, the data used in the study were only from one city, and cautions should be taken when generalizing the results to other geographic areas. In addition, we used ambient particulate matter from fixed site rather than individual exposure, which may result in measurement error, and the individual risk factors such as smoking, drinking and underlying diseases were unknown and not controlled in the study. Furthermore, as we used time series method to estimate the effects which was considered inappropriate to estimate long-term effects [32], it should be careful when analysing the long-term associations.

## Conclusions

In conclusion, our study provided a new insight into the disease burden due to COPD of ambient PM_2.5_ exposure. We found that PM_2.5_ had significant impacts on YLL due to COPD in Ningbo, China. Furthermore, the exposure-response curve was nonlinear and the associations were steeper at low levels and became flattened out at higher exposures. The elderly were susceptible population. Our findings highlight the importance and urgency of ambient PM_2.5_ control and protection of the vulnerable populations.

## Additional file

Additional file 1: Table S1. Life expectancy for Chinese population (averaged value for 2011-2015). **Table S2.** Threshold selection based on the Akaike Information Criterion value. **Table S3.** Sensitivity analyses of associations of PM_2.5_ and PM_10_ with YLL and ER of COPD deaths. **Figure S1.** Distribution of daily deaths and daily YLL of COPD in Ningbo, 2011-2015, China. **Figure S2.** Auto-correlation function for residuals of PM_2.5_ single pollutant models for YLL of COPD at lag04. **Figure S3.** Auto-correlation function for residuals of PM_2.5_ single pollutant models for deaths of COPD at lag04. **Figure S4.** Auto-correlation function for residuals of PM_10_ single pollutant models for YLL of COPD at lag04. **Figure S5.** Auto-correlation function for residuals of PM_10_ single pollutant models for deaths of COPD at lag04. (DOCX 21 kb)

## Abbreviations

ACF: Autocorrelation function
AIC: Akaike information criterion
CI: Confidence interval
COPD: Chronic obstructive pulmonary disease
ER: Excess risk
GAM: Generalized additive model
NO_2_: Nitrogen dioxide
PM_10_: Inhalable particulate matter
PM_2.5_: Fine particulate matter
SO_2_: Sulfur dioxide
WHO: World Health Organization
YLL: Years of life lost
