# Supplementary material for: The short term burden of ambient fine particulate matter on chronic obstructive pulmonary disease in Ningbo, China
Source: Environ Health. 2017 Jun 6;16:54. doi: 10.1186/s12940-017-0253-1 (PMC5461635; doi:10.1186/s12940-017-0253-1)
Supplement: Additional file 1: Table S1. — Life expectancy for Chinese population (averaged value for 2011-2015). Table S2. Threshold selection based on the Akaike Information Criterion value. Table S3. Sensitivity analyses of associations of PM2.5 and PM10 with YLL and ER of COPD deaths. Figure S1. Distribution of daily deaths and daily YLL of COPD in Ningbo, 2011-2015, China. Figure S2. Auto-correlation function for residuals of PM2.5 single pollutant models for YLL of COPD at lag04. Figure S3. Auto-correlation function for residuals of PM2.5 single pollutant models for deaths of COPD at lag04. Figure S4. Auto-correlation function for residuals of PM10 single pollutant models for YLL of COPD at lag04. Figure S5. Auto-correlation function for residuals of PM10 single pollutant models for deaths of COPD at lag04. (DOCX 21 kb) [file 12940_2017_253_MOESM1_ESM.docx]

Additional file 1

The short term burden of ambient fine particulate matter on chronic obstructive pulmonary disease in Ningbo, China

Table of contents

**Table S1** Life expectancy for Chinese population (averaged value for 2011-2015)

**Table S2** Threshold selection based on the Akaike Information Criterion value

**Table S3** Sensitivity analyses of associations of PM_2.5_ and PM_10_ with YLL and ER of COPD deaths

**Figure legends in Supplemental Material**

**Figure S1** Distribution of daily deaths and daily YLL of COPD in Ningbo, 2011-2015, China

**Figure S2** Auto-correlation function for residuals of PM_2.5_ single pollutant models for YLL of COPD at lag04

**Figure S3** Auto-correlation function for residuals of PM_2.5_ single pollutant models for deaths of COPD at lag04

**Figure S4** Auto-correlation function for residuals of PM_10_ single pollutant models for YLL of COPD at lag04

**Figure S5** Auto-correlation function for residuals of PM_10_ single pollutant models for deaths of COPD at lag04

**Table S1** Life expectancy for Chinese population (averaged value for 2011-2015)

(<http://apps.who.int/gho/data/view.main.60340?lang=en>)

| Age group | Life expectancy(year) | |
| --- | --- | --- |
|  | Female | Male |
| 0 | 77.2 | 74.14 |
| 1 | 76.96 | 74 |
| 5 | 73.12 | 70.16 |
| 10 | 68.22 | 65.26 |
| 15 | 63.32 | 60.36 |
| 20 | 58.42 | 55.46 |
| 25 | 53.52 | 50.62 |
| 30 | 48.66 | 45.82 |
| 35 | 43.82 | 41.04 |
| 40 | 39.04 | 36.32 |
| 45 | 34.3 | 31.62 |
| 50 | 29.64 | 27.02 |
| 55 | 25.04 | 22.58 |
| 60 | 20.7 | 18.38 |
| 65 | 16.66 | 14.62 |
| 70 | 13.06 | 11.32 |
| 75 | 9.96 | 8.64 |
| 80 | 7.44 | 6.62 |
| 85 | 5.48 | 4.94 |
| 90 | 4.16 | 3.72 |
| 95 | 3.24 | 2.9 |
| 100 | 2.8 | 2.36 |

**Table S2** Threshold selection based on the Akaike Information Criterion value

| AIC value | threshold(μg/m^3^) | AIC value | threshold(μg/m^3^) |
| --- | --- | --- | --- |
| 19327.91 | 100 | 19323.73 | 126 |
| 19327.96 | 101 | 19322.99 | 127 |
| 19327.97 | 102 | 19322.56 | 128 |
| 19327.98 | 103 | 19324.73 | 129 |
| 19327.96 | 104 | 19325.25 | 130 |
| 19327.9 | 105 | 19325.25 | 131 |
| 19327.9 | 106 | 19324.61 | 132 |
| 19327.95 | 107 | 19323.43 | 133 |
| 19327.88 | 108 | 19324.94 | 134 |
| 19327.88 | 109 | 19324.94 | 135 |
| 19327.96 | 110 | 19324.94 | 136 |
| 19326.96 | 111 | 19324.94 | 137 |
| 19326.14 | 112 | 19324.94 | 138 |
| 19324.86 | 113 | 19324.94 | 139 |
| 19325.02 | 114 | 19324.94 | 140 |
| 19325.02 | 115 | 19323.2 | 141 |
| 19325.26 | 116 | 19323.07 | 142 |
| 19325.66 | 117 | 19323.07 | 143 |
| 19325.66 | 118 | 19323.07 | 144 |
| 19327.54 | 119 | 19323.07 | 145 |
| 19327.68 | 120 | 19323.07 | 146 |
| 19326.89 | 121 | 19323.07 | 147 |
| 19323.45 | 122 | 19323.07 | 148 |
| 19323.45 | 123 | 19323.07 | 149 |
| 19323.45 | 124 | 19323.07 | 150 |
| 19323.73 | 125 |  |  |

**Table S3** Sensitivity analyses of associations of PM_2.5_ and PM_10_ with YLL and ER of COPD deaths

| Parameter changed in the model | Years of life lost (95%CI) | Excess risk (95%CI) |
| --- | --- | --- |
| PM_2.5_  Relative humidity(4 of degrees of freedom) | 0.93(0.18, 1.68) | 1.34(0.54, 2.14) |
| Temperature (4 of degrees of freedom) | 0.91(0.16, 1.66) | 1.32(0.52,2.12) |
| Time per year (6 of degrees of freedom) | 0.89(0.15,1.63) | 1.30(0.51, 2.09) |
| Time per year (8 of degrees of freedom) | 0.96(0.20, 1.72) | 1.39(0.58, 2.20) |
| PM_10_ |  |  |
| Relative humidity(4 of degrees of freedom) | 0.83(0.31, 1.34) | 1.08(0.52,1.64) |
| Temperature(4 of degrees of freedom) | 0.82(0.30,1.33) | 1.08(0.51,1.64) |
| Time per year(6 of degrees of freedom) | 0.78(0.27,1.30) | 1.07(0.51, 1.62) |
| Time per year(8 of degrees of freedom) | 0.82(0.30, 1.34) | 1.11(0.54, 1.68) |

Associations were presented with 10 μg/m^3^ increase in PM_2.5_ and PM_10_ at lag04 (moving average concentrations from day 0 to day4)_._ Results were adjusted for seasonality, day of the week, temperature and relative humidity. Single pollutant models were used. Data were collected from Ningbo, China, 2011-2015.
